# Welcome note International Journal of Integrated Care (IJIC)

**DOI:** 10.5334/ijic.1039

**Published:** 2012-09-04

**Authors:** Lourdes Ferrer

**Affiliations:** UK

On behalf of the Editorial Board of the *International Journal of Integrated Care* (*IJIC*) it gives me great pleasure to welcome you to the Republic of San Marino for the 12th Annual Conference on Integrated Care.

“*May you live in interesting times*” is a well-known quote that has been used and interpreted both as an old Chinese curse or as a dynamic reality in International Law. This conference today is an opportunity to shape this reality by learning from each other’s experiences, both practical and academic, and to get a deeper understanding of the dynamic multi-dimensional field that is integrated care.

This year’s conference programme in San Marino is rich in interesting presentations and poster papers and we look forward to publishing the abstracts in *IJIC*’s annual conference supplement. We cordially invite you to write-up your policy analyses, academic research and/or innovative case studies and submit them formally or informally to the Journal.

During the last 12 years of existence, *IJIC* has consolidated itself as the leading international scientific journal in its field. *IJIC* remains a pioneer in providing an open access and e-based publication that is widely-read. *IJIC*, as a result, is also highly responsive enabling one of the shortest periods from article submission to publication that there is—25 weeks in 2010. In 2011, *IJIC*’s unofficial Impact Factor rose to 1.75—a good number for a specialist journal—and an application for an official impact factor is in progress.

Since its innovative start, *IJIC* and its related annual conferences have delighted us with important developments in the knowledge and practice of integrated care. This collective learning experience has been possible because of the leading role of Prof. Guus Schrijvers, our sponsors at the Julius Centre, and our helpful publishers—IGITUR—at the University Library Utrecht. It has also been steered over the years by a highly committed and experienced Board of Editors, all of whom have given their time voluntarily, and supported by a range of excellent authors and reviewers. But, especially, it has been a very rich experience due to the on-going support of our more than 1500 monthly readers from more than 25 countries.

By the end of 2012, the new *International Foundation of Integrated Care* (*IFIC*) will replace the Julius Centre as the journal sponsor. IFIC is an ambitious and exciting initiative with a growing portfolio of initiatives including: a new World Congress; an Integrated Care Knowledge Centre; a new ‘high tech’ website; a range of educational courses; and a core partner in international research projects.

*IFIC* is an evolving organization and you are welcome to help contribute to growing portfolio of work and also its planning, decision-making and development processes. To learn more about *IJIC* and *IFIC*, please attend our workshop session (4.3) on day 2. It is indeed a very interesting time for all of us who wish to join this initiative and contribute to shape the work of *IJIC* and *IFIC* in the future.

Finally, we are extremely grateful to the conference organizers and the scientific committee for their hard work in putting together such a fine event. Special thanks go to Paolo Pasini and his colleagues in San Marino for agreeing to be such welcoming hosts.

We wish all of you an interesting and effective conference.

